# Effect of chemotherapeutic drugs and cytochalasin B on tunneling nanotubes in U87 MG cells

**DOI:** 10.1186/s12885-025-15204-7

**Published:** 2025-11-04

**Authors:** Nicole Matejka, Jessica Neubauer, Judith Reindl

**Affiliations:** 1 Institute for Applied Physics and Measurement Technology, University of the Bundeswehr Munich, Neubiberg, Germany; 2 Department of Physics, University of Oslo, Oslo, Norway

**Keywords:** Glioblastoma, Tunneling nanotubes, Chemotherapy, Therapy resistance, Drugs, Cellular communication

## Abstract

Intercellular communication in the tumor microenvironment has a major impact on therapeutic outcomes. In particular, highly aggressive cancers such as glioblastoma benefit from efficient cellular communication by developing resistance to therapy or by infiltrating surrounding tissue to escape radiotherapy. Tiny membrane tunnels, such as tunneling nanotubes (TNTs), can serve as an effective communication tool for tumor cells. With these structures, the cells can build a large network that allows them to react and adapt to their environment as quickly as possible, thereby combating the therapy. In this study, we investigated the effects of chemotherapeutic agents on TNT networks in U87 MG cells to determine if they affect the number of TNTs and, consequently, possible therapeutic outcomes. The effect of drugs on TNT stability may be related to their effects on actin and tubulin, which are known stabilizers of membrane structures. Therefore, we also analyzed the cytoskeletal content of TNTs in U87 MG cells and examined the effect of the actin polymerization inhibitor cytochalasin B (CytoB) on TNT formation and networks. CytoB reduced the number of TNTs in this study, suggesting that actin, as a cytoskeletal component, is required for developing TNT networks. In contrast, the chemotherapeutic agents temozolomide (TMZ) and cytarabine (AraC) do not affect TNTs and actin content. As TNT networks are closely linked to increased therapy resistance of certain tumors, we believe that the lack of suppression of TNTs is a major problem in current glioblastoma therapy and should be a target for further research in the future.

**Supplementary Information:**

The online version contains supplementary material available at 10.1186/s12885-025-15204-7.

## Background

Tunneling nanotubes (TNTs) are tiny membrane connections between distant cells with diameters in the nanometer range. These fine connections, which can be more than 100 μm in length, are an efficient cellular communication tool for the direct and rapid transfer of information or cargo. They were first described in rat pheochromocytoma PC12 cells in 2004 [1]. Since their discovery, they have been found in several other cell lines, both cancerous and normal, in 2D cell culture as well as in tissue. TNTs have been shown to transport several types of cargo, including calcium signals, death signals, cell organelles, viruses, and more [2]. Research on TNTs has revealed that these membrane tunnels display considerable morphological and functional diversity across different cell types. However, TNTs are strongly suspected to play a crucial role in cancer treatment due to their advantageous properties in facilitating rapid exchange between cells, thus ensuring good adaptability of the cells to their environment. Especially, in cancers with high intra-heterogeneity, the interaction and cooperation of different subclones of cells can lead to therapy failure by creating a therapy-resistant microenvironment [3]. It has been shown that TNTs can form large resistant networks and transfer cargoes such as mitochondria, p-glycoproteins, or miRNAs that promote therapy resistance [4–10]. On the other hand, several reports demonstrate that nanoparticles can also be transferred via TNTs and, therefore, one could also think about using TNTs as a drug delivery system [11–14]. TNTs may provide new ideas for cancer treatment, especially for very aggressive cancers such as glioblastoma, where the need for new therapeutic approaches is high. Glioblastoma is the most common and malignant human brain tumor with a very poor prognosis of fewer than 15 months median survival [15, 16]. The aggressiveness of this type of cancer is due to its high heterogeneity [3], large genomic alterations [17, 18], high rate of infiltration into the surrounding healthy tissue [19], and strong ability to develop resistance to therapy [20–23]. The standard treatment for glioblastoma is to first surgically remove as much of the tumor mass as possible, followed by radiation and concurrent chemotherapy with the drug temozolomide (TMZ) [21, 24, 25]. Glioblastoma is characterized by its infiltrative growth pattern into surrounding healthy brain tissue [19, 26], challenging complete surgical resection. During radiation therapy, infiltrating tumor cells within healthy tissue regions may receive suboptimal radiation doses, enabling them to survive and continue to grow. Therefore, chemotherapy represents an essential complementary treatment modality with the potential to target disseminated tumor cells more comprehensively. For the chemotherapy of glioblastoma, alkylating agents such as TMZ are commonly used [21]. However, very often the tumor recurs close to the primary tumor with increased resistance to therapy [19]. The interaction of individual cells, particularly the effective cell communication enabled by TNTs, can contribute to the problem of developing resistance to therapy. The hypothesis that TNTs may be a desirable therapeutic target has been widely discussed in the literature [2, 27–34]. However, up today the knowledge about TNTs is very limited. Their great diversity among different cell types makes it even more difficult to identify key points about these membrane structures. Thus, their relationship to cancer and their prospects for new therapeutic approaches remain largely unknown [2]. To shed more light on the role of TNTs, it is necessary to understand how communication via TNTs is affected by different therapeutic circumstances, such as different types of irradiation or the use of different chemotherapeutic agents.

This study investigates the effect of chemotherapeutic drugs on cellular communication via TNTs in the U87 MG glioblastoma cell line. We used the drug TMZ, commonly used in glioblastoma therapy, and selected cytarabine (AraC) as a second chemotherapeutic agent to analyze its effect on cellular communication via TNTs for two reasons. First, AraC has been reported to inhibit TNTs in leukemic cells after 24 h of incubation at a concentration of 1 µM [35, 36], and second, AraC is also being studied in the context of glioblastoma therapy, as this chemotherapeutic agent also crosses the blood-brain barrier [37–39]. Both chosen chemotherapy drugs disrupt DNA replication. TMZ causes DNA methylation [40] and AraC acts as an incorrect DNA building block [41]. We also used cytochalasin B (CytoB) to investigate whether TNTs can be inhibited in U87 MG cells and to examine the role of actin in TNTs. CytoB is an actin polymerization inhibitor [42] and TNT inhibition by CytoB has been shown in many other cell lines [43–45], thus its use allows conclusions about the role of actin in TNTs in addition to live-cell microscopy images. In addition, we performed live-cell confocal microscopy of the cytoskeleton of the TNTs to check whether tubulin and/or actin are present in the TNTs. We preferred live-cell imaging because cell fixation causes many TNT breaks and therefore distorts the TNT network, as shown in previous studies [7, 46].

## Methods

### Cell culture

The human glioblastoma cell line U87 MG (ATCC, Manassas, VA, USA, HTB-14) was kindly provided by the Institute of Radiation Medicine (Helmholtz Zentrum München GmbH, 85764 Neuherberg, Germany). Cells were cultured in DMEM, high glucose medium (Sigma-Aldrich, St. Louis, MO, USA, D6429) supplemented with 10% FBS (Sigma-Aldrich, St. Louis, MO, USA, F7524) and 1% penicillin/streptavidin Sigma-Aldrich, St. Louis, MO, USA, P4333), and maintained at 37 °C in a controlled atmosphere of 5% CO_2_ and 95% humidity.

### Confocal live-cell imaging system

Confocal live-cell imaging was performed using a Leica TCS SP8 3X confocal microscope (Leica Microsystems CMS GmbH, Mannheim, Germany) equipped with a live-cell imaging unit consisting of a climate box and CO_2_ supply with a humidifier (“The Cube & the Box”, Life Imaging Services GmbH, Basel, Switzerland). Cells were imaged under live-cell conditions of 37 °C and 5% CO_2_.

### TNT identification

For TNT visualization, the cell membrane was stained. Membrane connections were identified as TNTs when two separate cells are connected by a membrane connection smaller than 1 μm in diameter, and if the connection is located above the substrate. The last criterion was determined by acquiring z-stacks for imaging and evaluating the TNTs using 3D images rather than maximum projections. The characteristics of the TNTs of U87 MG, including their morphology, lifetime, formation, and dimensions, as well as the optimized methodology for studying the nature of the TNTs by suitable confocal live-cell imaging, were thoroughly described by Matejka et al. [46].

### Statistical analysis

The two-sample t-test (GraphPad Software, QuickCalcs, Boston, MA, USA, version 9) and two-way ANOVA with repeated measures, followed by Bonferroni post-hoc tests (IBM SPSS Statistics, International Business Machines Corporation (IBM), Armonk, NY, USA, version 29), were used to resolve significant differences. A *P*-value of ≤ 0.05 was considered statistically significant.

### Quantification of the cytoskeletal content of TNTs in U87 MG cells

#### Cell seeding

For cytoskeleton analysis, the cells were seeded in a µ-Dish 35 mm (Ibidi GmbH, Gräfelfing, Germany, 81158) at a seeding density of 65,000 cells/ml (2 ml volume per dish) the day before the experiment. The dishes have a glass bottom with a thickness of (170 ± 15) µm and are therefore well suited for high-resolution microscopy.

#### Cytoskeleton and membrane staining

The SiR-tubulin kit (Tebubio GmbH, Offenbach, Germany, SC002) and the SiR700-actin kit (Tebubio GmbH, Offenbach, Germany, SC013) were used to label the cytoskeleton of U87 MG cells. A dye concentration of 750 nM was used to label actin and tubulin with the application of 5 µM verapamil (an efflux pump inhibitor included in the kits). The cells were incubated in the staining solution for at least 3 h at 37 °C, 95% humidity, and 5% CO_2_. For TNT visualization, the cellular membrane was stained either with a 1.5X CellMask™ Green plasma membrane staining solution (Thermo Fisher Scientific Inc., Waltham, MA, USA, C3760) or Vybrant™ DiO labeling solution (Thermo Fisher Scientific Inc., Waltham, MA, USA, V22886). The cells were stained with CellMask™ Plasma Membrane Stains using a 1.5X staining solution dissolved in growth medium. After incubating for 10 min at 37 °C with 95% humidity and 5% CO₂, the cells were washed three times with growth medium. To stain the cell membranes with DiO, the cells were incubated with a 5 µM staining solution dissolved in medium at 37 °C, 95% humidity, and 5% CO_2_ for 20 min. Then, the cells were washed three times with growth medium for 10 min at 37 °C, 95% humidity, and 5% CO_2_.

#### Confocal live-cell imaging

For imaging, a 100× oil objective (Leica Microsystems CMS GmbH, Mannheim, Germany) with a high numerical aperture of 1.4 and the halogen- and fluorescence-free immersion oil Immersol™ 518 F/37 °C from Zeiss (Carl Zeiss Microscopy Deutschland GmbH, Oberkochen, Germany, 444970-9010-000) with a refractive index of 1.518 at 37 °C were used. The cells were imaged in DMEM medium without phenol red (Thermo Fisher Scientific, Inc., Waltham, MA, USA; 21063029). This medium significantly reduces background noise.

The co-staining images were acquired sequentially. The first sequence captured membrane staining, and the second sequence captured double cytoskeleton staining. The sequence was switched between stacks to ensure rapid acquisition and to minimize cell movement during acquisition. In addition, the scan speed was set to 600 Hz, and bidirectional scanning was used. The CellMask™ Green plasma membrane dye was excited at a wavelength of 522 nm. The signal of the CellMask™ Green plasma membrane dye was detected in a range of 530–583 nm with a hybrid detector (HyD, Leica Microsystems CMS GmbH, Mannheim, Germany). To capture the DiO signal, the dye was excited at a wavelength of 484 nm and detected within the range of 501–583 nm. The same wavelength of 630 nm was used to excite the SiR-tubulin and SiR700-actin probes, and two HyD were used simultaneously for detection. One detector was set to a range of 650–700 nm to detect the signal of SiR-tubulin, and the other detector was set to a range of 710–750 nm to detect the signal of SiR700-actin. The pixel size was either 40–80 nm, and the z-step was either 160–400 nm, depending on whether STED imaging was attempted. Unfortunately, STED imaging was unsuccessful because the cytoskeleton labeling yielded insufficient signal for STED imaging. Therefore, only confocal images were used for the analysis.

#### Image Deconvolution and 3D visualization

The analysis of the cytoskeletal content of the TNTs was performed using the raw data. However, for image visualization, the acquired images were deconvolved using the Huygens Professional Deconvolution software (Scientific Volume Imaging B.V., Hilversum, The Netherlands, version 23.04). Based on the microscopy parameters (metadata) used, a theoretical point spread function (PSF) was calculated by the software. The deconvolution was carried out using an iterative algorithm of the Classic Maximum Likelihood Estimation (CMLE) as established in our lab for several approaches [46, 47]. The deconvolution process was performed with an SNR value of three and an automatic background estimation (Estimation mode: Lowest) with an area radius of 0.7 μm.

The 3D visualizations were generated using the Simulated Fluorescence Process (SFP) rendering method with the Huygens SFP Volume Renderer. This software is available with the Huygens Professional Deconvolution program from Scientific Volume Imaging B.V., Hilversum, Netherlands (version 23.04).

### Investigation of the effects of drugs on TNT networks

#### Cell seeding

For TNT network analysis, the cells were seeded in a CELLview cell culture dish 35 × 10 mm with four compartments (Greiner Bio-One GmbH, Frickenhausen, Germany, 627870) at a seeding density of 55,000 cells/ml (1 ml volume per well; 28,947 cells/cm^2^). The dishes have a glass bottom with a thickness of (170 ± 15) µm and are therefore well suited for high-resolution microscopy.

#### Membrane staining

The cellular membrane was stained similarly to the experiments that quantified the cytoskeletal content of TNTs. However, an orange variant of CellMask™ plasma membrane stain (Thermo Fisher Scientific Inc., Waltham, MA, USA, C10045) was used because it is more photostable [46].

#### Drug treatment

After staining, different concentrations of TMZ (Sigma-Aldrich, St. Louis, MO, USA, T2577), AraC (Sigma-Aldrich, St. Louis, MO, USA, C1768), or CytoB (Sigma-Aldrich, St. Louis, MO, USA, C6762) were used to treat the cells. For TMZ, concentrations of 1 µM, 10 µM, and 100 µM were chosen. For AraC, concentrations of 0.1 µM, 1 µM, 10 µM, and 100 µM were selected, and for CytoB, concentrations of 0.05 µM, 0.5 µM, and 5 µM were investigated. The concentration range was deliberately chosen to be wide, ensuring that no effect would be missed by accidentally selecting a concentration that is too low or too high. Publications using TMZ [48–51], AraC [35], or CytoB [43–45, 52] to study TNTs, toxicity, or radiation effects were used as a reference point for the concentration ranges. The freshness and stability of the drugs were ensured by using newly purchased stock solutions that were aliquoted to minimize repeated freeze-thaw cycles. The maximum concentrations of dimethyl sulfoxide (DMSO) used were 0.5% for the highest concentrtion of CytoB (5 µM) and 0.2% for the highest concentrtion of TMZ (100 µM).

#### Acquiring confocal live-cell videos

Immediately after drug addition, the sample was mounted on the confocal microscope, and live-cell videos were acquired. The time required to prepare and check the microscope settings for the videos was kept to a minimum. In total, defining the areas to be imaged, creating the focal planes for each imaging area, and setting the remaining microscope parameters took less than 50 min. A mean time ± standard deviation of (36 ± 7) minutes elapsed between the addition of drugs and the start of imaging. By using dishes with four separate compartments, it was possible to image four different treatments simultaneously, ensuring ideal comparability. In most cases, a control and the treatment of three different concentrations of a substance were imaged simultaneously. One area was imaged per treatment, and each treatment was performed at least three times.

For imaging, a 63x oil objective (Leica Microsystems CMS GmbH, Mannheim, Germany) with a high numerical aperture of 1.4 and the halogen- and fluorescence-free immersion oil Immersol™ 518 F/37 °C from Zeiss (Carl Zeiss Microscopy Deutschland GmbH, Oberkochen, Germany, 444970-9010-000) with a refractive index of 1.518 at 37 °C were used. The CellMask™ Orange plasma membrane stain was excited with a wavelength of 554 nm, and the detection range of the hybrid detector (HyD) was set to 567–635 nm. A 3 × 3 mosaic image with 10% overlap was acquired, resulting in a final merged field of view of approximately 510 μm x 515 μm with a mean number of 40 ± 13 cells at the beginning of the video. All videos were acquired as 20-step z-stacks with a step size of 400 nm, resulting in an acquired height of 7.6 μm. The pixel size was 80 nm, and the cells were scanned bidirectionally at 600 Hz to reduce cell stress. Imaging was performed for up to 24 h with a time interval of 30 min. To keep the cells in focus throughout the video, a 9-point rectangular focus map per area was defined, and the autofocus provided by the Leica Application Suite X (LAS X) software (Leica Microsystems CMS GmbH, Mannheim, Germany, version 3.5.6) of the microscope was used.

#### TNT network analysis

The TNT network was analyzed as described by Matejka et al. [7] with raw images. Briefly, we selected specific time points of the videos and evaluated the TNTs per hand while scrolling through the z-stack and looking at each cell separately. During the analysis, we distinguished between two types of connections: simple connections, consisting of one to two TNTs, and complex connections, consisting of three or more TNTs. There were two reasons for this distinction. First, for evaluation purposes, the individual TNTs of very dense connections consisting of five or more TNTs were so closely packed that they were indistinguishable and therefore not countable, as reported in Matejka et al. [7]. Second, the number of TNTs between two cells can serve as an indicator of the strength or efficiency of a connection because the more TNTs there are between two cells, the more cargo can be transported simultaneously. Thus, according to our definition, a complex connection could be more efficient than a simple connection. A further subclassification, e.g., by TNT number alone, was not recommended because connections containing exactly two, three, or four TNTs were rarely present.

In the analysis, cells with one or more connections of any type are referred to as connected cells (e.g., cells having TNTs). Furthermore, as an indicator of the complexity of the network, connected cells that have connections to at least two other cells are referred to as highly connected cells and were also counted. The TNT network was graded based on the number of connected cells, connections per cell (regardless of connection type), percentage of complex connections, and number of highly connected cells. For each field, the number of cells was recorded, and the TNT networks were quantified using percentages of connected and highly connected cells as well as the number of connections per cell (connections divided by cell count), thereby normalizing for field-to-field variation in cell density.

We quantified TNT networks at 1, 3, 10, and 24 h after adding the drug and compared them with those of untreated control cells. The time points were chosen to encompass potential stages of cellular communication following drug addition. We were guided by our prior observation of a pronounced TNT network response 6 h after α-particle irradiation [7]. Since TNTs are formed within minutes and have a lifespan of about 90 min, we focused our attention on the first few hours after the drug was added. Selecting this time frame also prevents density-related artifacts caused by late cytotoxicity.

## Results

### Cytoskeletal content of TNTs

We performed live-cell microscopy to examine the cytoskeletal content of the TNTs in U87 MG cells. For this purpose, we simultaneously labeled the plasma membrane, tubulin, and actin of the cells as described in the methods section. A total of 85 TNTs were analyzed for cytoskeletal content in three independent experiments.

**Fig. 1:**
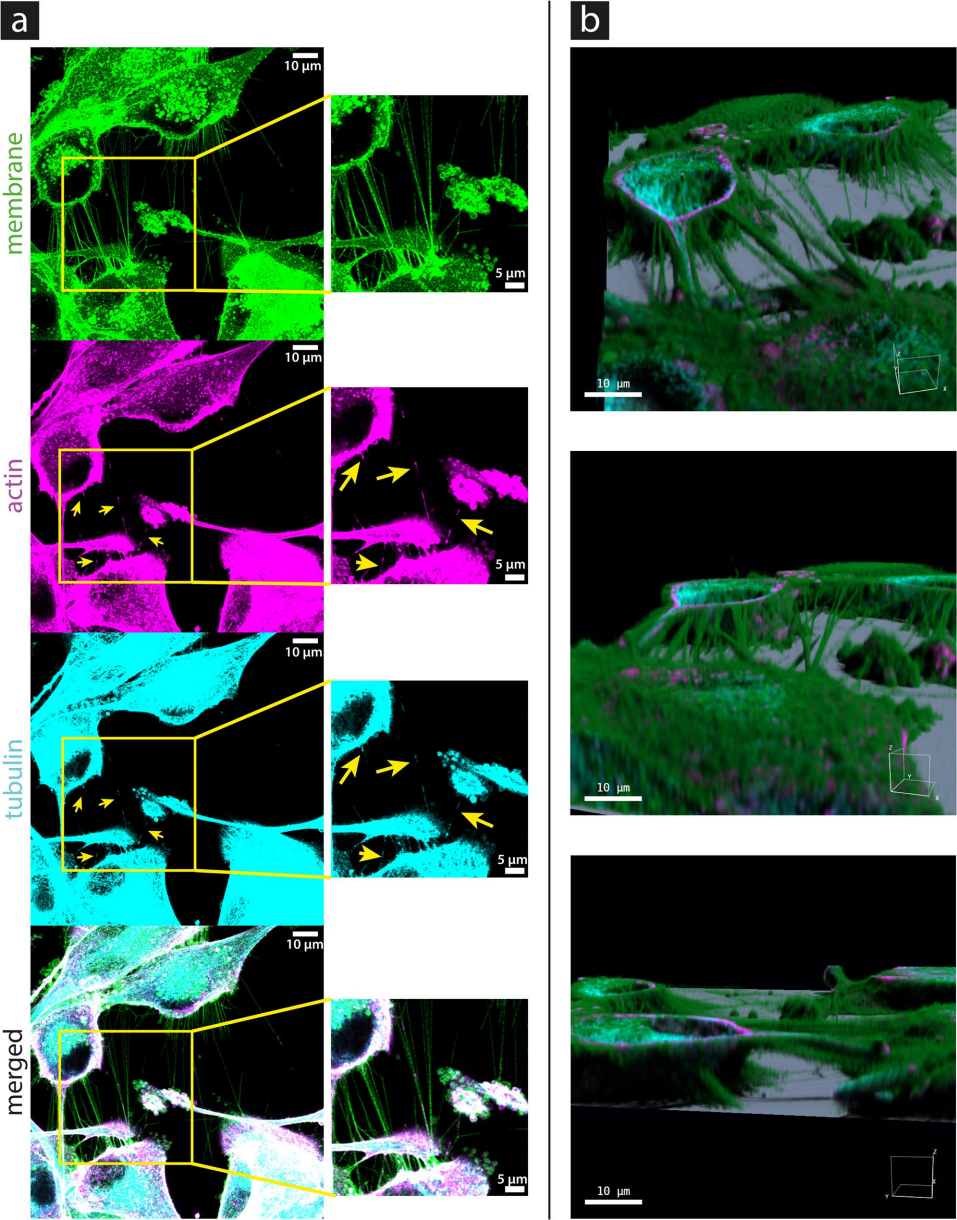
**a** Exemplary deconvolved live-cell confocal images (maximum projections) and rendered 3D images (SFP volume rendering) of U87 MG cells with labeled membrane (green), actin (magenta), and tubulin cytoskeleton (cyan). TNTs are visible as straight, thin membrane structures that connect cells in the membrane channel. Other fine membrane structures that do not connect cells are filopodia. They end nowhere and are visible at the border of the upper cell, for example. Cell nuclei are recognizable as black ellipses because they contain neither lipids nor cytoskeletons. The four yellow arrows mark the positions where actin and tubulin are visible in four different TNTs. In **b**, SFP volume rendering images are shown, providing different perspectives of the entire acquired z-stack. The 3D images show that the TNTs are located above the substrate. More 3D images are provided in the supplementary material. The scale bar is 10 μm long in the overview and 3D images, and 5 μm long in the magnified images located in the center

**Fig. 2:**
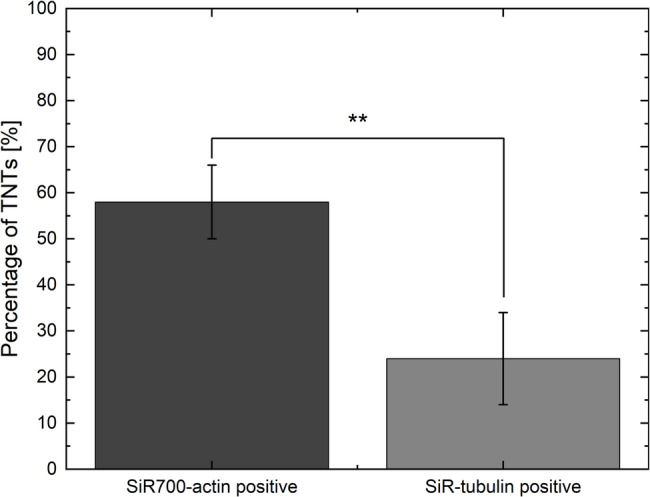
Analysis of the cytoskeletal content of TNTs in U87 MG cells. Actin was found more often than tubulin in TNTs. According to the two-sample t-test (*N* = 3), a *P*-value of *P* = 0.01 is marked by **. Means ± SD of three independent experiments are shown

The microscopy images show that both actin and tubulin signals are not always visible in the tiny TNTs. Figure 1 (a) shows exemplary confocal images revealing the fragmentary presence of actin and tubulin in the TNTs of U87 MG cells. The full z-stack, including both raw and deconvolved images, is provided in Supplementary Figs. 1 and 2, respectively. The location of the TNTs above the substrate can be seen in the 3D images (SFP volume rendering) shown in Fig. 1 (b). More 3D images from different perspectives are provided in the supplementary material (see Supplementary Figs. 3–5). Actin is detectable in (58 ± 8; mean ± SD) % of the TNTs and tubulin in (24 ± 10) % of the TNTs (see Fig. 2). There are significantly (two-sample t-test (*N* = 3): *P* = 0.01) fewer TNTs containing tubulin. The analysis revealed that all tubulin-containing TNTs also contained actin. Therefore, (24 ± 10) % of the TNTs contained both cytoskeletal content, and (34 ± 12) % of the TNTs only contained actin. The cytoskeleton content in TNTs is much lower compared to the rest of the cytoskeleton located in the cell interior; therefore, image processing such as contrast enhancement is necessary to make the cytoskeleton content in TNTs visible (see Fig. 1). The reduced fluorescence in the TNTs indicates that there is a reduced presence of cytoskeletal components in the TNTs compared to the cell interior. The signal strength of the cytoskeletal content varies between TNTs and along the length of the TNTs. In the analysis, a TNT was considered actin- or tubulin-positive if a signal could be detected anywhere along the TNT.

### TNT network alteration using CytoB

In our study, we analyzed the effect of CytoB on the TNT networks of U87 MG cells. We labeled the membrane of U87 MG cells using the CellMask™ Orange plasma membrane stain, then we added the drug dissolved in normal growth media at concentrations of 0.05 µM, 0.5 µM, and 5 µM, and acquired confocal live-cell videos for up to 10 h after drug addition. Each condition was performed at least three times with a minimum of one sample, and the average was determined. In our experiment, we used four-compartment cell culture dishes, which allow microscopy of four different concentrations simultaneously, ensuring good comparability.

**Fig. 3:**
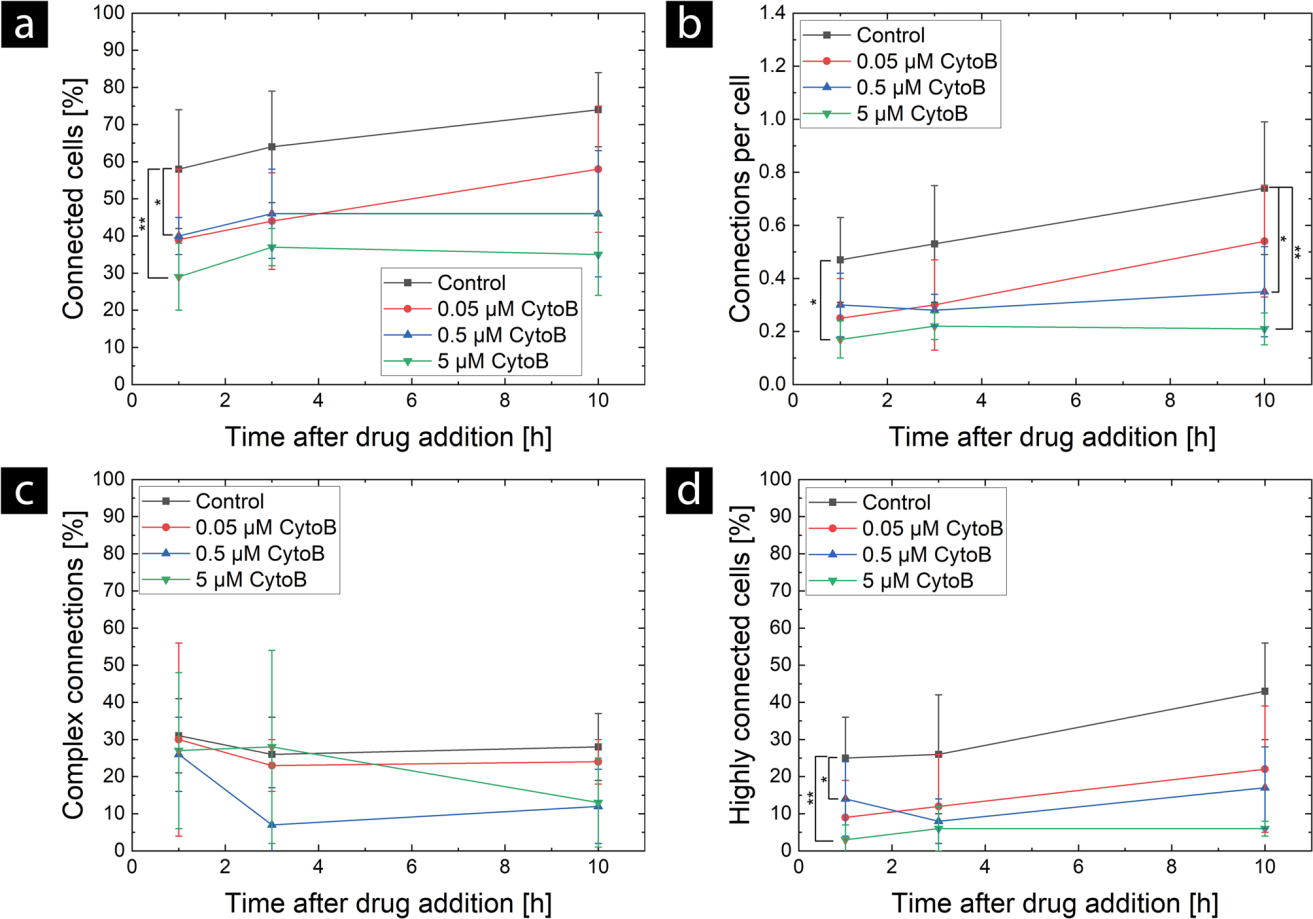
Effect of CytoB at different concentrations on TNT networks in U87 MG cells. The mean values ± standard deviations of (**a**) the number of connected cells, (**b**) the number of connections per cell, (**c**) the percentage of complex connections, and (**d**) the number of highly connected cells are shown. The mean values and standard deviations for each CytoB-treated group were determined from at least three independent experiments (*N* = 4 for 0.05 µM CytoB and 0.5 µM CytoB; *N* = 3 for 5 µM CytoB), each with one sample. The mean value of the control group was determined using data from eleven independent experiments (*N* = 11), each with one sample. Significant differences were identified using two-way ANOVA with repeated measures, followed by Bonferroni post-hoc tests. *P*-values of ≤ 0.05, or ≤ 0.01 are indicated by *, or **, respectively. No interaction effects were identified for the analysis of the number of connected cells (**a**) and the number of highly connected cells (**d**). Here, the *P*-values correspond to the means over time

CytoB causes a reduction in TNT connections as the number of connected cells (Fig. 3 (a)), as well as the number of connections per cell (Fig. 3 (b)), decreases with the addition of CytoB. The effect becomes stronger at higher concentrations of CytoB. The effect is present at the earliest time point of 1 h, suggesting a rapid mechanism of network disruption. At the highest concentration, only (29 ± 9) % of the cells are connected at 1 h after addition, which is a significant (Two-sample t-test; *P* = 0.0120) twofold reduction compared to the control samples (58 ± 16) %. In the control samples, the percentage of connected cells increases to (74 ± 10) %. In contrast, in all treated samples, the network is not able to recover, which can be expected since the drug is present throughout the imaging period. After 10 h, the reduction is significant for all CytoB concentrations added (Two-sample t-test; 0.05 µM: *P* = 0.0396; 0.5 µM: *P* = 0.0015; 5 µM: *P* < 0.001) in comparison with the control groups. Similar trends were found for connections per cell, where a significant reduction was found for all time points for the 5 µM treatment, while a slight recovery was found for the 0.05 µM treatment throughout the measured period. In addition to the number of connected cells and the number of connections per cell, we also evaluated the complexity of the TNT network by determining the number of highly connected cells and the proportion of complex connections, as shown in Fig. 3 (c) and (d). Highly connected cells are cells that are connected to more than one other cell, and complex connections are connections that consist of at least three individual TNTs or more. CytoB treatment causes a significant (Two-sample t-test; *P* = 0.0061) 5-fold reduction in the number of highly connected cells (Fig. 3 (d)) for the earliest time point (control: (25 ± 11) %, 5 µM: (3 ± 4) %). In contrast to the control, samples treated with 0.5 µM and 5 µM are not able to increase the number of highly connected cells. The 0.05 µM samples show a slight recovery. Furthermore, the two higher concentrations tend to suppress complex connections at late time points. It can be concluded that CytoB reduces the complexity of the TNT network at concentrations as high as 0.5 µM. This effect becomes stronger with higher concentrations of CytoB. At the lowest concentration, an immediate effect is seen, which is at least partially reversed at later time points.

In addition to the two-sample t-tests, we performed statistical analysis using two-way ANOVA with repeated measures, followed by Bonferroni post-hoc tests to identify significant differences. Significant differences were found among the groups regarding the number of connected cells, connections per cell, and highly connected cells. No interaction effects were identified regarding the number of connected cells or highly connected cells. However, the two-way ANOVA analysis of connections per cell revealed an interaction effect. Therefore, Bonferroni post-hoc tests were performed for each time point separately. The resulting *P*-values from the Bonferroni post-hoc tests are shown in Table 1. These statistical analyses revealed significant differences in the number of connected and highly connected cells when comparing the controls with the two higher concentrations of CytoB. Significant differences were identified in the analysis of connections per cell for the comparison of the control with the two higher concentrations of CytoB at the 10-hour time point and additionally at the 1-hour time point for the highest concentration of CytoB (5 µM).

**Table 1:** Resulting *P*-values of performed bonferroni post-hoc tests

Comparison	Connected cells	Highly connected cells	Connections per cell at 1 h	Connections per cell at 3 h	Connections per cell at 10 h
Control – 0.05 µM CytoB	*P* = 0.087	*P* = 0.143	*P* = 0.093	*P* = 0.227	*P* = 0.333
Control – 0.5 µM CytoB	*P* = 0.03	*P* = 0.026	*P* = 0.327	*P* = 0.136	*P* = 0.012
Control – 5 µM CytoB	*P* = 0.003	*P* = 0.004	*P* = 0.025	*P* = 0.082	*P* = 0.003

### Effect of TMZ and AraC on the TNT network

After this proof that certain drugs can sustainably alter the network, we were interested in whether certain chemotherapeutic drugs might be able to induce similar effects. We chose TMZ as a standard-of-care drug for glioblastoma treatment and AraC as a promising new drug that has already been used to modify the TNT network in other cancer cell lines. The experimental design, procedure, and replication were the same as for CytoB treatment. TMZ concentrations of 1 µM, 10 µM, and 100 µM and AraC concentrations of 0.1 µM, 1 µM, 10 µM, and 100 µM were used.

**Fig. 4:**
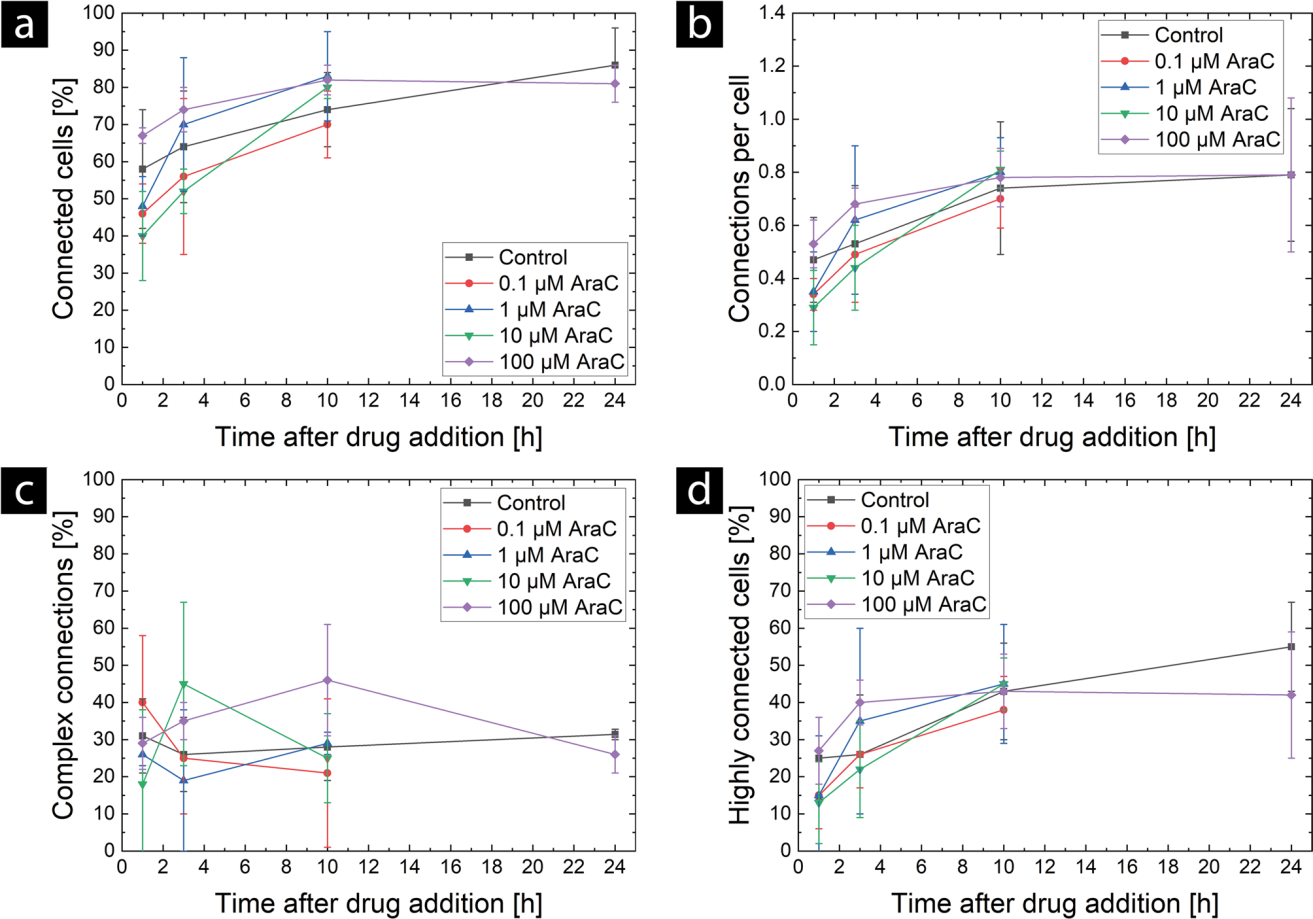
Effect of AraC at different concentrations on TNT networks in U87 MG cells. The mean values ± standard deviations of (**a**) the number of connected cells, (**b**) the number of connections per cell, (**c**) the percentage of complex connections, and (**d**) the number of highly connected cells are shown. The mean values and standard deviations for groups at 0.1 µM, 1 µM, and 10 µM were determined from three independent experiments (*N* = 3), each with one sample. The mean for the control group at the 1-, 3-, and 10-hour time points was determined using data from eleven independent experiments (*N* = 11) with one sample each. For the 24-hour time point, the mean value for the control group was calculated using data from five independent experiments (*N* = 5), each with one sample. The mean value for the 100 µM AraC group was calculated based on two independent experiments (*N* = 2), each with two or three samples. Two-way ANOVA with repeated measures revealed no significant differences

**Fig. 5:**
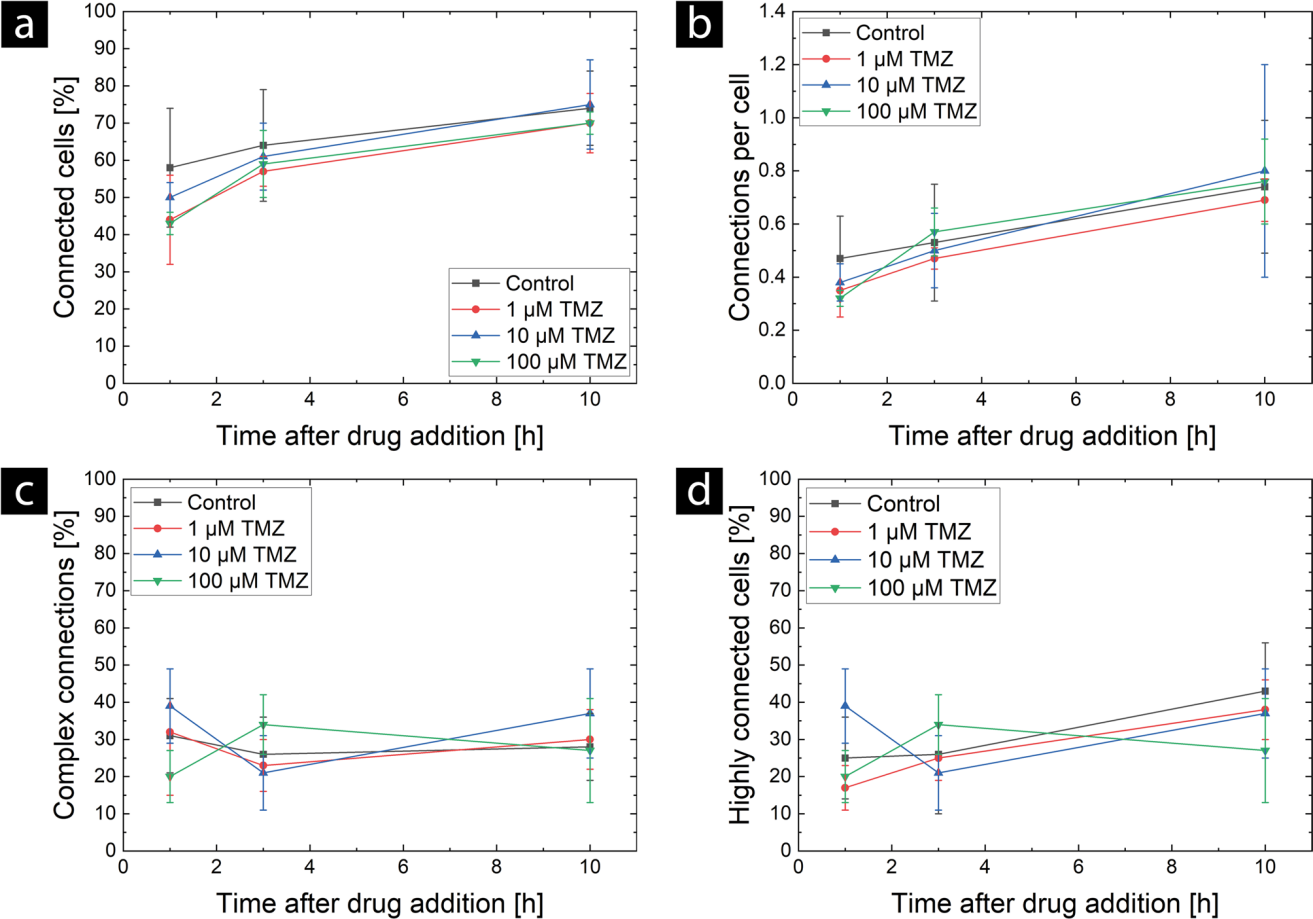
Effect of TMZ at different concentrations on TNT networks in U87 MG cells. The mean values and standard deviations are shown for the following: (**a**) number of connected cells, (**b**) number of connections per cell, (**c**) percentage of complex connections, and (**d**) number of highly connected cells. The mean values and standard deviations for each TMZ-treated group were determined from three independent experiments (*N* = 3), each with one sample. The mean value of the control group was determined using data from eleven independent experiments (*N* = 11), each with one sample. Two-way ANOVA with repeated measures revealed no significant differences

AraC and TMZ do not affect the TNT network in U87 MG cells (see Figs. 4 and 5). No significant difference was observed in either the number of connected cells (Fig. 4 (a) and Fig. 5 (a)) or the number of connections per cell (Fig. 4 (b) and Fig. 5 (b)) according to two-way ANOVA with repeated measures. At the beginning of imaging, the fraction of connected cells varied between (40 ± 12) % and (67.0 ± 2.1) % with no dependence on chemotherapeutic treatment, with a higher variability in the AraC treatment. After 10 h of imaging, this fraction increases to 70% to 83% for all measured conditions. At the 10-hour time point, the fraction of connected cells has reached a plateau, and no changes are visible in the samples evaluated for up to 24 h (control and AraC 100 µM). The analysis of the connections per cell shows the same trends. Again, the AraC treatment shows a higher variability (0.29 ± 0.14 to 0.53 ± 0.09) compared to the TMZ treatment (0.32 ± 0.03 to 0.38 ± 0.07) at the earliest time point and reaches a similar plateau at 10 h, which is in good agreement with the measurements of the control samples. Therefore, we suggest that AraC may cause an increase in the natural variability of the TNT formation at early time points, but does not have a specific and lasting effect on the TNT networks themselves. As the chemotherapeutic agents are present in the cells throughout the whole measurement, a specific effect should be visible in the first hours, since in a previous study we could show that TNT formation occurs within minutes, and the lifetime of TNTs in U87 MG cells is about 90 min [46]. In addition, no clear alterations in network complexity were found for either drug at any concentration. The results are shown in Fig. 4 (c and d) for AraC and Fig. 5 (c and d) for TMZ. Two spikes are visible in the time course of the proportion of complex connections: one for the 10 µM AraC treatment at 3 h and one for the 100 µM AraC treatment at 10 h. According to the two-sample t-test, these spikes differ significantly from the control group (*P* = 0.0418 and *P* = 0.0194, respectively). However, two-way ANOVA with repeated measures revealed no significant differences for either TMZ or AraC treatments regarding the proportion of complex connections or the number of highly connected cells.

## Discussion

The first objective of this study was to clarify the cytoskeleton content in TNT connections between U87 MG cells in vitro. We found that actin is more present compared to tubulin, but both proteins show only presence in a fraction of TNTs at low concentration. We suspect that the concentrations are so low that the signal might disappear in the background and is barely measurable. Together with their small diameter and large length, this suggests that TNTs are fragile membrane structures with low cytoplasmic content. In this study, we also tested whether CytoB reduces the number of TNTs in glioblastoma cells, as this has been reported for several other cell types and is commonly used to inhibit TNTs [43–45]. Our results show that TNTs in U87 MG glioblastoma cells are also suppressed by CytoB, already in the first hour of treatment for all concentrations, and a sustainable effect for concentrations of 0.5 µM and 5 µM. The effect becomes stronger the higher the concentration of CytoB. Therefore, our results are consistent with other reports on TNTs and indicate that actin is important for these membrane structures. The inhibition of TNTs by an actin polymerization inhibitor such as CytoB would suggest that actin plays an important role in TNT formation, stability, or possible transport mechanisms along TNTs, which has been investigated in many studies in different cell types [43, 53–56]. However, further research is needed to gain a deeper understanding of the role of actin or other cytoskeletal components in TNTs in glioblastoma. The role of tubulin, which is responsible for transporting a variety of cargo, especially needs to be explored in glioblastoma cells. Inhibitors of tubulin, such as nocodazole, could be used for this purpose. Different components of the cytoskeleton likely mediate distinct transport mechanisms within TNTs. For example, it has been observed that the types of cargo transferred in human macrophages are affected by the cytoskeletal content of the TNTs [57]. Intracellular organelles, such as endosomes, lysosomes, and mitochondria, were specifically exchanged through thicker, tubulin-rich TNTs. Bacteria, on the other hand, were seen to move only by riding along the surface of thinner, actin-based TNTs. Furthermore, bidirectional cargo transport has been observed in TNTs containing tubulin, suggesting that tubulin-associated molecular motors facilitate this process [54]. Additionally, TNTs containing tubulin may be more stable than those consisting solely of F-actin due to the increased stiffness provided by tubulin filaments [58].

We believe that CytoB is not a suitable TNT inhibitor for further research into the functions of TNTs because it has a strong effect on the cytoskeleton, which is undoubtedly necessary for cell morphology, migration, and proliferation, as CytoB is also used as a cytokinesis blocker in the micronucleus technique [59]. Therefore, the use of CytoB on its own would strongly influence cellular behavior, and this could lead to false conclusions about cellular communication via TNTs. Consequently, there is still a lack of TNT inhibitors for further TNT research in glioblastoma.

In addition to these results, we also recognize that the TNT networks exhibit large natural fluctuations. The large error bars (standard deviations) in Figs. 3 and 4, and 5 show that the individual measurements vary considerably. We would like to emphasize that the control curves (black lines) are the result of eleven independent measurements, which were taken to better characterize baseline fluctuations in TNT networks. Even here, however, the standard deviations are large and not significantly lower than in the other measurements, for which three independent experiments were conducted. Therefore, we assume that TNT networks are naturally highly variable. This high natural variability could mask small effects. Nevertheless, the reduction of the TNT network as a result of CytoB treatment is well visible, and therefore, it is possible to measure such influences in TNT networks.

The second main objective of this study was to investigate the effect of chemotherapeutic agents on TNT networks in glioblastoma cells. Our results show that the chemotherapeutic agents TMZ and AraC do not affect the TNT networks in U87 MG cells. TMZ is commonly used, and AraC is also being investigated in glioblastoma therapy [37, 38]. Both drugs can overcome the brain-blood barrier, which is a major criterion for the treatment of glioblastoma [60]. Both TMZ and AraC disrupt the DNA replication in the tumor cell, thereby blocking DNA repair and cell division [40, 41]. AraC is commonly used to treat acute myeloid leukemia (AML) [61, 62]. TNTs are also suspected of promoting chemoresistance in the treatment of AML and are therefore also being studied in the context of this disease [35, 36]. It was found that AraC reduces the number of TNTs in primary AML cells and AML cell lines [36]. These results indicate that treating AML with AraC also effectively suppresses the cellular communication of the tumor cells, suggesting that AraC as a chemotherapeutic agent in treating AML is promising. The results of this study indicate that AraC does not appear to effectively suppress the formation of TNT networks in U87 MG cells. Therefore, a synergistic effect resulting from the disruption of DNA replication in the primarily treated cells, combined with the inhibition of TNT-mediated communication, seems unlikely under these conditions.

Our results additionally show that TMZ, the most commonly used chemotherapeutic agent in glioblastoma treatment, does not affect TNTs in U87 MG glioblastoma cells. A study by Valdebenito and colleagues even demonstrates that TMZ enhances the formation of membrane connections at larger sizes [49]. However, their analysis was performed at a lower magnification compared to the present study, which likely captured other membrane structures that are significantly larger than TNTs. Nevertheless, it is still not known how membrane connections such as epithelial bridges, microtubes, or TNTs are related to each other, which are all involved in cellular communication in glioblastoma cells [2, 4, 33]. Therefore, we can conclude that TMZ does not appear to prevent cellular communication through membrane tunnels in either microtubes or TNTs. This could potentially lead to therapy resistance and a poor prognosis.

In our opinion, these results suggest that the missing downregulation of cellular communication along membrane structures such as TNTs, microtubes, or epithelial bridges by TMZ might be one of the main problems in the current treatment of glioblastoma. However, this hypothesis needs to be proven by further research on the actual role of cellular communication through membrane structures such as TNTs in cancer progression. TNTs in glioblastoma cells are formed as cells are moving apart from each other [46]. In addition, TNTs are often found at the migration front of cells [32, 46]. Thus, it seems that cell migration and cell communication via TNTs are related, and therefore, TNTs may promote cell invasiveness. To prove this hypothesis, further live-cell studies of TNTs are needed.

In addition, our findings underlie the diversity of TNTs in different cell types. AraC reduces the number of TNTs in AML cells, but we see no effect of AraC on TNT networks in U87 MG cells. This shows that we need to be aware that our findings about TNTs in one cell type do not necessarily apply to TNTs in other cell types. One potential explanation for the observed lack of suppression of TNTs in U87 MG cells by AraC could be the distinct types of TNT formation observed between AML cells and glioblastoma cells. TNTs are formed by filopodia growth among AML cells [36] and by cell movement among glioblastoma cells [46]. These observations suggest that the mechanisms underlying TNT formation may differ between AML and glioblastoma cells. Moreover, the genomes of AML cells and glioblastoma cells may differ, which could also account for the varied responses of their TNT networks and their reactions to treatment.

There was no significant difference in the number of cells per field between the treated and control groups. The number of cells in the control group increased slightly over time (from 40 ± 11 cells after 1 h to 62 ± 17 cells after 24 h), whereas the number of cells in the treated group remained almost constant, indicating cytotoxic effects (see Supplementary Fig. 6). This suggests that the lack of observed effects is not artificially caused by a decrease in cell number due to cell detachment. Additionally, we used a wide range of concentrations, and the drugs were present in the medium throughout the whole experiment. Therefore, the lack of observed effects probably did not result from using an inadequate drug concentration or exposure time. However, the combination of different treatments is an interesting point that wasn’t addressed in this study. Both AraC and TMZ disrupt DNA replication. Therefore, combining these drugs with interventions that induce DNA damage, such as radiation therapy, could result in a different TNT network response than using these interventions alone.

Of course, this study is not without limitations. First, it uses a single cell line, which does not adequately represent the heterogeneity of glioblastoma. However, the primary objective of this study was to offer fundamental mechanistic insights into TNT-mediated cellular communication, rather than to directly investigate clinical correlations. Further research is needed to validate the findings of this study, including experiments with patient-derived glioblastoma cells that better reflect glioblastoma heterogeneity. Furthermore, we did not investigate potential long-term or immediate effects occurring within the 36 ± 7 min required to transfer cells to the microscope and prepare them for imaging. Since TNTs are fragile structures, we suspect that sample preparation procedures, such as washing steps or replacing the culture medium with a drug-containing medium, could affect the TNT network. As a result, detecting early effects could be very challenging. In our experience, the CellMask plasma membrane stain provides sufficient signal intensity for up to 24 h [46]. However, for imaging periods exceeding 24 h, we recommend refreshing the labeling to maintain adequate signal quality. Note that refreshing the labeling will also influence TNT network measurements, particularly when repeatedly imaging the same cells over an extended period. In addition, the high natural variability observed in the TNT networks in this study could potentially mask small effects. This challenge proposes investigating more robust methods that can include a greater number of replicates for the treatment groups. Finally, this study did not investigate functional transfer along TNTs in U87 MG cells. Further research is needed to determine which cargoes can be exchanged and contribute to the development of therapy resistance in glioblastoma cells. In addition, the question of whether the functionality of the TNTs is affected by the drugs must be addressed.

## Conclusion

In conclusion, this study shows that the chemotherapeutics TMZ and AraC do not suppress TNT networks in U87 MG glioblastoma cells. We hypothesize that the lack of consistent inhibition of cellular communication across TNTs in glioblastoma cells, such as U87 MG cells, by currently used chemotherapeutic agents may contribute to therapy resistance. Although our results suggest that AraC and TMZ do not significantly impact TNT formation, this finding alone is insufficient to confirm that TNT-mediated intercellular exchange is a mechanism by which tumor cells survive and resist treatment. Additional experiments are needed to confirm this hypothesis. For example, co-cultivating untreated cells with cells treated with chemotherapeutic agents, or conducting functional experiments involving genetic knockdowns of TNT-related genes, could demonstrate how TNTs promote chemotherapy resistance. This would underscore the importance of researching therapeutic strategies that could directly target TNTs. Furthermore, our results show that the actin polymerizing inhibitor CytoB reduces the number of TNTs in U87 MG glioblastoma cells. Therefore, we can conclude that actin polymerization is crucial for TNTs in glioblastoma cells. However, CytoB strongly affects the cytoskeleton of the cells and therefore has a major impact on cell behavior and appearance. Thus, we suggest that CytoB is not suitable for TNT inhibition for further TNT research. We believe that TNTs play a crucial role in the interaction of cells in the tumor microenvironment. Nevertheless, the involvement of TNTs in cancer progression remains unknown. Further studies are necessary to determine the clinical relevance of TNT-mediated cellular communication, particularly by investigating correlations between enhanced TNT networks and patient prognosis, treatment resistance, or other clinically meaningful outcomes. By learning more about these membrane structures, which do not appear to be affected by currently used chemotherapeutics, we may be able to find new therapeutic approaches and prevent the development of therapy resistance in glioblastoma therapy.

## Supplementary Information

Supplementary Material 1.

Supplementary Material 2.

Supplementary Material 3.

Supplementary Material 4.

Supplementary Material 5.

Supplementary Material 6.

## Data Availability

The datasets used and/or analyzed during the current study are available from the corresponding author upon reasonable request.

## Abbreviations

TNT: Tunneling nanotube
TNTs: Tunneling nanotubes
TMZ: Temozolomide
AraC: Cytarabine
CytoB: Cytochalasin B
DMSO: Dimethyl sulfoxide
AML: Acute myeloid leukemia
