# Supplementary figures and images for: Effect of chemotherapeutic drugs and cytochalasin B on tunneling nanotubes in U87 MG cells
Source: BMC Cancer. 2025 Nov 4;25:1709. doi: 10.1186/s12885-025-15204-7 (PMC12587739; doi:10.1186/s12885-025-15204-7)

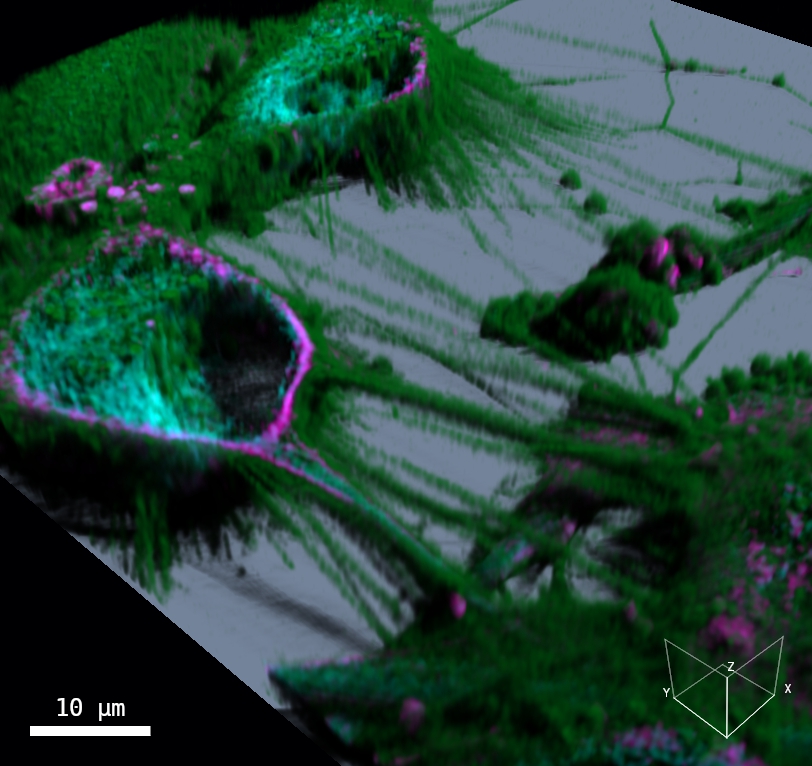

Supplement: Supplementary file 3 — Supplementary Material 3. [file 12885_2025_15204_MOESM3_ESM.jpg]

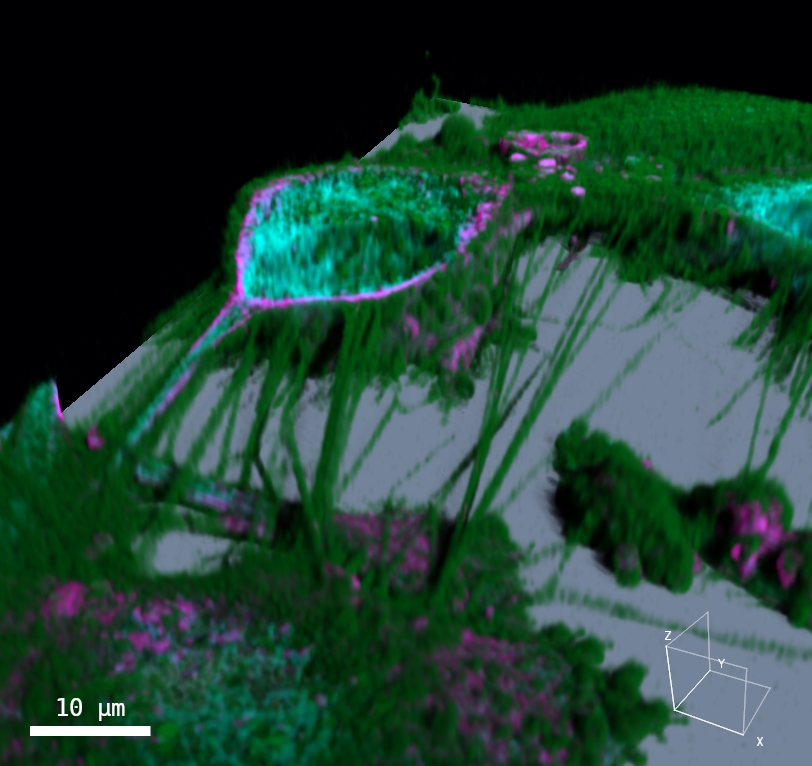

Supplement: Supplementary file 4 — Supplementary Material 4. [file 12885_2025_15204_MOESM4_ESM.jpg]

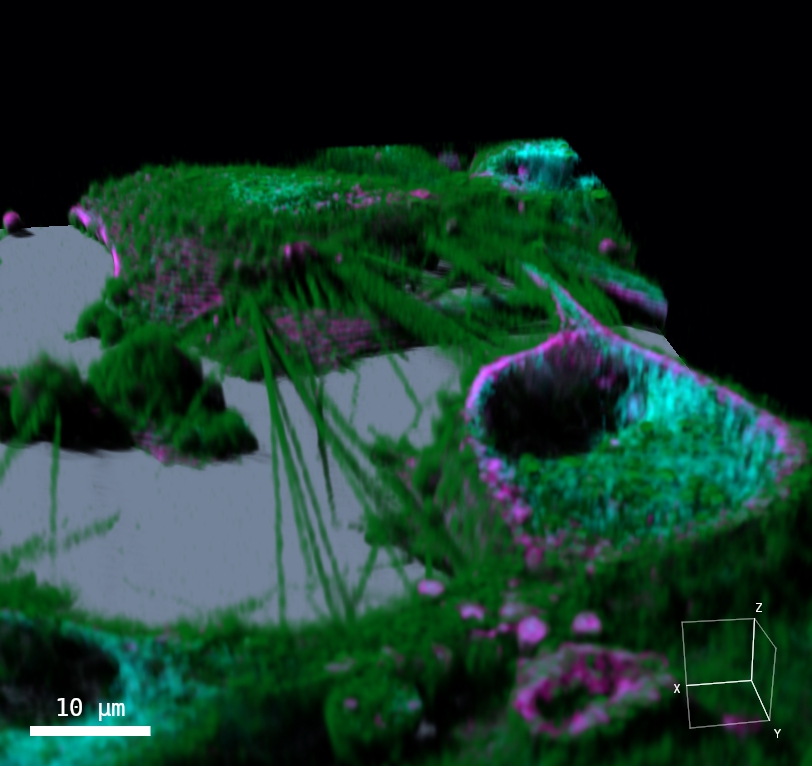

Supplement: Supplementary file 5 — Supplementary Material 5. [file 12885_2025_15204_MOESM5_ESM.jpg]

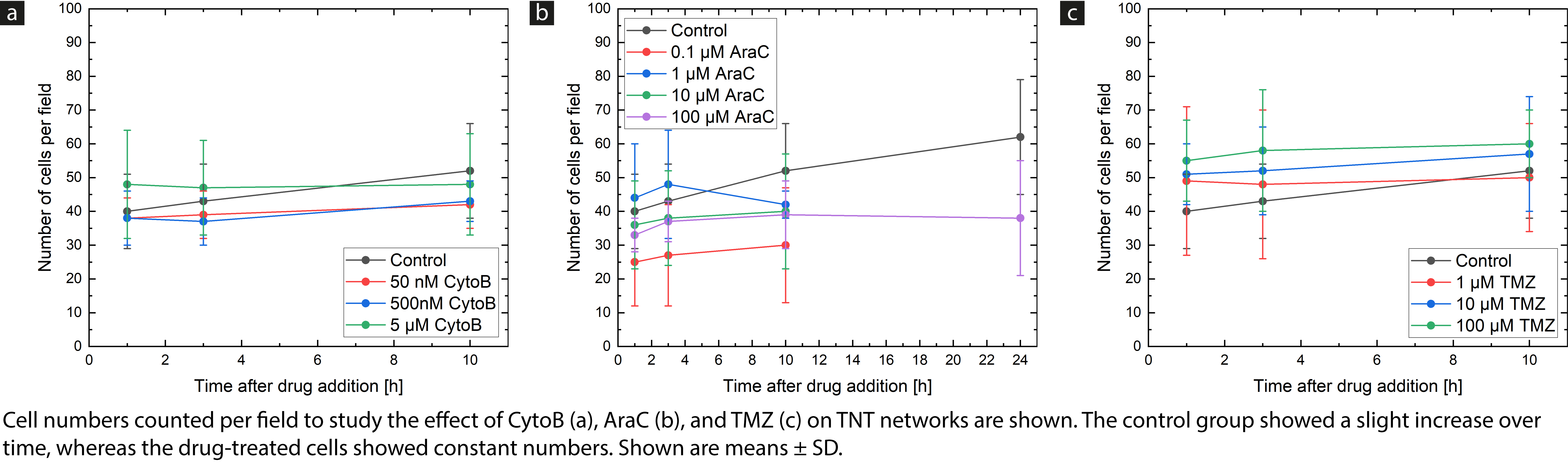

Supplement: Supplementary file 6 — Supplementary Material 6. [file 12885_2025_15204_MOESM6_ESM.tif]
